# Compressive Behavior of Some Balls Manufactured by 3D Printing from Ceramic–Polymer Composite Materials

**DOI:** 10.3390/mi15010150

**Published:** 2024-01-19

**Authors:** Adelina Hrițuc, Vasile Ermolai, Andrei Marius Mihalache, Liviu Andrușcă, Oana Dodun, Gheorghe Nagîț, Marius Andrei Boca, Laurențiu Slătineanu

**Affiliations:** 1 Department of Machine Manufacturing Technology, “Gheorghe Asachi” Technical University of Iași, 700050 Iași, Romania; vasile.ermolai@student.tuiasi.ro (V.E.); andrei.mihalache@tuiasi.ro (A.M.M.); oanad@tcm.tuiasi.ro (O.D.); nagit@tcm.tuiasi.ro (G.N.); marius-andrei.boca@student.tuiasi.ro (M.A.B.); slati@tcm.tuiasi.ro (L.S.); 2 Department of Mechanical Engineering, “Gheorghe Asachi” Technical University of Iași, 700050 Iași, Romania; andrusca.liviu@yahoo.com

**Keywords:** ceramic composite materials, polymer matrix, finite-element modeling, balls, compression, pottery clay, terracotta, concrete, granite, gravimetric analysis, influencing factors

## Abstract

It is known that ceramic–polymer composite materials can be used to manufacture spherical bodies in the category of balls. Since balls are frequently subjected to compression loads, the paper presents some research results on the compression behavior of balls made of ceramic composite materials with a polymer matrix. The mathematical model of the pressure variation inside the balls highlights the existence of maximum values in the areas of contact with other parts. Experimental research was carried out on balls with a diameter of 20 mm, manufactured by 3D printing from four ceramic–polymer composite materials with a polymer matrix: pottery clay, terracotta, concrete, and granite. The same ceramic–polymer composite material was used, but different dyes were added to it. A gravimetric analysis revealed similar behavior of the four materials upon controlled heating. Through the mathematical processing of the experimental results obtained by compression tests, empirical mathematical models of the power-type function type were determined. These models highlight the influence exerted by different factors on the force at which the initiation of cracks in the ball materials occurs. The decisive influence of the infill factor on the size of the force at which the cracking of the balls begins was found.

## 1. Introduction

Composite materials are made up of a metallic or non-metallic mass, called a matrix, reinforced with resistance elements, such as short or very short fibers, long fibers, fabrics, felt, etc. Although known in distinct forms for a relatively long time, composite materials have seen an obvious expansion of their use in recent decades. This expansion was possible due to improved mechanical properties and new technologies for manufacturing parts from composite materials. Currently, composite materials are used to manufacture vehicles, sports equipment, the space industry, etc. It is estimated that about 75% of composite parts are currently based on polymer matrices [1]. A ceramic material is a hard and usually brittle material made by shaping and firing an inorganic metallic material. In frequent cases, ceramic materials are made from mixtures of clay, earthen elements, powders, and water. Bodies with different shapes are prepared from such mixtures, which are later fired in the oven. Apart from high hardness and high fragility, ceramic materials can be corrosion-resistant, wear-resistant, oxidation-resistant, chemically stable, refractory, thermal insulators, electrical insulators, and non-magnetic, but prone to thermal shocks. Given such properties, it was normal to use ceramic materials to manufacture parts made of composite materials with a polymer matrix, for which ceramic materials could at least partially provide some of the previously mentioned advantages [2].

Thus, some ceramic composite materials use matrices of ceramic or polymer materials. There are, therefore, technologies in which a ceramic matrix incorporating polymeric materials is required, with the result that, subsequently, through appropriate treatments, the polymeric material is removed or at least partially transformed into a material with specific properties of a ceramic material. Thus, Miyazaki et al. considered that the mechanical mixing of bioactive fillers in an organic polymer matrix could lead to the manufacture of composite materials. Such composite materials could interact better with the human body in the case of various medical applications [3]. Dziadek et al. analyzed the properties of some bio-degradable ceramic–polymer composites used in medical applications [4]. They appreciated that it is possible to manufacture parts from polymer–ceramic composites with properties that can be controlled to obtain parts from materials better adapted to the requirements of their use.

One of the trends in the field of manufacturing technologies was directing research efforts toward a deeper knowledge of the properties of composite materials with ceramic or polymer matrices and the identification of technologies for manufacturing parts from such materials. Thus, a group of manufacturing processes frequently used in the last few decades are 3D printing processes. Manufacturing parts from ceramic composite materials with a ceramic or polymer matrix was possible. Singh et al. studied the possibilities of manufacturing thick ferroelectric–ceramic composite films that can be used for efficient energy storage [5]. They appreciated that such materials could be used as power-density electronic elements in green energy. The results of research on the identification of a 3D printing process for manufacturing parts from a polymer–ceramic composite material capable of ensuring antibacterial effects were communicated by Marin et al. [6,7]. They considered that such parts could be used in the manufacture of components that come into contact with pathogens, for example, covers for mobile phones, mice, pens, air filters, and tool handles. The problem of the possible influence of the anisotropy of a ceramic composite material containing methacrylate and tricalcium phosphate particles from samples manufactured by 3D printing on the mechanical properties of that material was studied by Stögerer et al. [8]. They concluded that, for the conditions in which the research was carried out, an isotropic behavior could not be highlighted from the point of view of the mechanical properties of the investigated material. Tactile sensor arrays made of a laminar polymer–ceramic composite were studied by Idhaiam [9]. The research followed the behavior of a single-layer sensor architecture and a laminar polymer–HfO_2_ composite material from multiple points of view, aiming to optimize the constructive solution of the sensor.

The possibilities of using some ceramic materials for restorative dentistry were investigated by Shi et al. [10]. They found that resin-based ceramics can be used instead of other ceramic materials due to their properties. In a paper on dental ceramics, new materials, and processing methods used in dental restorations, Silva et al. concluded that composite materials processed via CAD-CAM could be interesting. Such materials present intermediate mechanical properties between ceramics and polymers but can be milled and polished more easily [11]. The limits of an additive manufacturing method of silicon oxide ceramic matrix composite test samples were investigated by O’Masto et al. [12]. They used a digital light projection printer to photopolymerize a siloxane-based preceramic resin that included inert ceramic reinforcement. They concluded that the investigated process allows for the free fabrication of high-performance components made of a ceramic matrix composite. A polymer–ceramic composite material for dental implant applications capable of withstanding high mechanical stress and wear was proposed and researched by Hodásová et al. [13]. They found that the copolymer infiltrated among the ceramic filaments can act as a mechanical stabilizer and adhesion promoter. The so-called slurry-based technologies use ceramic and polymer materials to manufacture parts through 3D ceramic printing [2,14]. The experimental results showed that the particle sizes significantly influence some parameters that characterize the manufacturing conditions of 3D printing ceramic materials. Okzan et al. considered the possibilities of manufacturing by 3D printing some components from ceramic materials of turbine blades [15]. They have developed a technology that allows manufacturing in complex economic conditions using cost-effective LCD printers. Different monomer mixtures were used to identify variants that could avoid crack propagation during subsequent sintering. Research results on the compression behavior of hollow spherical bodies from polylactic acid were presented in the work [16]. The compression of spheres from different wood categories manufactured using a tubular tool was addressed in the work [17].

It can be noted that the main research directions in the field of manufacturing parts from ceramic materials with a polymer matrix by 3D printing have focused on aspects related to the physical–mechanical properties of the materials of the parts [4,5,9,10], improving the manufacturing technologies of such parts [6,7,11,12,18,19,20], the influence of different factors on the material properties of parts manufactured by 3D printing [2,8,13,14,15], and the identification of new possibilities of using the respective materials [3,5,9,10,11,13,15].

The analysis of the results reached in the field of manufacturing and use of parts made of ceramic composite materials highlighted the existence of wide possibilities for obtaining a varied range with different properties from such materials. This paper formulated the problem of studying the compression stress behavior of some ball test samples manufactured from ceramic–polymer composites by 3D printing. Using the finite-element method should facilitate obtaining some initial information on the behavior of ball test samples under compression mechanical stresses. For this purpose, modeling of the processes that develop in parts shaped like balls made of a ceramic composite material with a polymer matrix when these parts are subjected to compression was undertaken using the finite-element method. It was appreciated that, by using gravimetric analysis, additional information can also be obtained regarding the behavior of ceramic–polymer composites during the 3D printing process. Later, it was possible to determine some empirical mathematical models capable of highlighting the influence of some factors related to the 3D printing process on the compressive strength of the materials incorporated in the ball test samples.

## 2. Materials and Methods

### 2.1. Spherical Bodies Subjected to Compression

The behavior under compressive stresses of some balls manufactured from ceramic–polymer composites by 3D printing was taken into account.

From a geometric point of view, a ball is a geometric body bounded by a spherical surface. The sphere is understood as the set of points located at the same distance from a point considered the center of the sphere.

Throughout history, spherical objects have been used for various purposes. The most well-known use of spherical bodies is rolling bodies in rolling bearings. However, applications are also based on the static use of spherical bodies. For example, in the case of some different technical structures, spherical bodies made of ceramic materials may be encountered, subject to static, quasi-static, or dynamic compression stresses [20,21,22,23].

**Figure 1:**
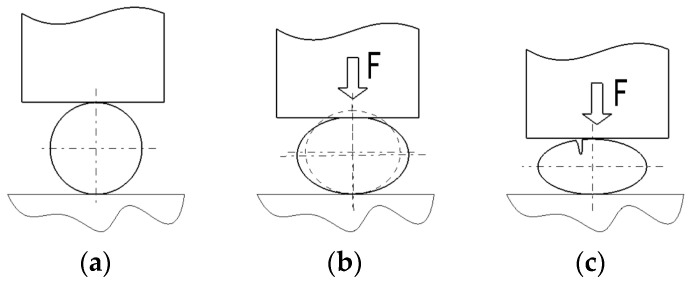
Behavior of the ball under force *F*: (**a**) initial situation; (**b**) ball compression; (**c**) the appearance of a first crack in the compressed ball.

The case of a spherical body placed on a hard surface and subjected to compression forces, *F*, through the flat surface of a pressure part (Figure 1a) was considered. Due to the resistance properties of the materials of such spherical bodies, it is expected that, initially, under the applied compression stresses, an elastic deformation will take place, leading to some deviations from the initial spherical shape into several zones of the original ball. The new body will be characterized by a lower height than the initial diameter, while the diameter corresponding to the middle area of the spherical body will increase (Figure 1b). Suppose the force exerted on the ball exceeds the elastic deformation limit of the ball’s material. In that case, the appearance of some first cracks is expected (Figure 1c) so that, later, by increasing the size of the cracks, the actual breakage will occur in several zones of the original ball.

The spherical shape of the test sample results in a variation in the pressure in the sample material, from a maximum value in the upper and lower areas of the ball, along a dimension, *h.* This dimension, *h*, provides information about the distance from the center of the ball to a surface parallel with an equatorial plane perpendicular to the direction of the axial force, *F*. In this equatorial plane, the pressure has a minimum value.

**Figure 2:**
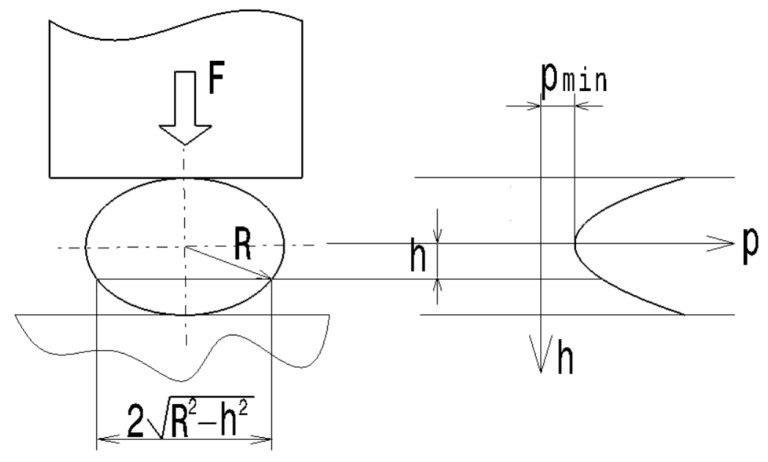
The pressure variation in the ball of radius *R* along a direction parallel to the direction of the compressive force, *F*.

The variation in the pressure, *p*, along the direction of the compression force, *F*, exerted on the ball of radius *R* can be determined as a ratio between the magnitude of the force, *F*, and the surface area of the sphere in a plane perpendicular to the direction of force *F* and located at a distance, *h*, from the sphere center (Figure 2). In a plane parallel to the equatorial plane and at a distance, *h*, from this equatorial plane, the radius, *r*, of the resulting circular surface determined by sectioning the sphere can be determined using the relation:

(1) $$r=\sqrt{R^{2}-h^{2}}$$

A minimum pressure value corresponds to a distance, *h* = 0, where the pressure, *p*, will correspond to the relationships:

(2) $$p=\frac{F}{\pi r^{2}}=\frac{F}{\pi \left(R^{2}-h^{2}\right)},$$

where *F* is the force exerted on the ball and *R* is the radius of the ball (Figure 2).

**Figure 3:**
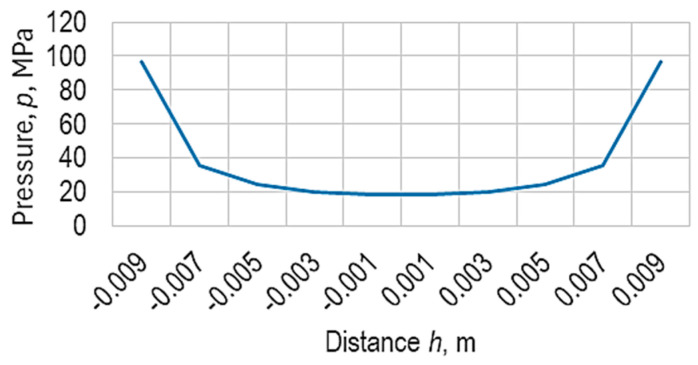
Pressure variation in the case of a ball radius, *R* = 1 mm, along a direction parallel to the direction of the compression force, *F* = 5750 N, according to Equation (2).

As an example, assuming a value of the force *F* = 5750 N (corresponding to one of the values of *F* at which the first crack appeared in the case of a ball made of the granite-type material the pressure, *p*, variation along a direction parallel to the direction of the axial force *F* can be observed in Figure 3. More detailed explanation will be provided in the experimental part on the research. The observation of how the crack appeared was performed visually and practically by the force–deformation diagrams developed using the computer program of the tensile–compression testing machine. At the time of the crack, there was a sudden drop in the magnitude of the force, *F*, applied to the ball.

As previously mentioned, ceramic materials are more fragile than ordinary polymeric materials. This means that they accept only fairly small elastic deformations without breaking. Such materials may be characterized by the acceptance of some elastoplastic deformations greater than those of a ball made exclusively of a ceramic material. However, in the present case, the balls are made of a composite material with a polymer matrix and particles of a ceramic material that are uniformly distributed in the polymer matrix.

Figure 3 shows that the maximum pressure values will be reached in the upper and lower areas of the ball, where there is the highest probability of an initial crack. However, the balls were manufactured by 3D printing. In the lower area of the ball, where the printing process started, the balls have a small, flat surface due to the contact of the molten material with the table of the printing equipment. Such a situation contributes to the appearance of a deviation from the spherical shape in the lower area of the ball. On the other hand, the balls may present a small protrusion in the upper area where the printing process has ended. This situation leads to lower values of the surface on which the compressive force, *F*, is applied or higher values of the pressure, *p*, in the upper areas of the balls. Consequently, the first cracks are expected to appear in the upper areas of the balls.

In the case of products made by 3D printing using an FFF-type process (fused filament fabrication), some characteristics of the surfaces of the printed parts (roughness and surface accuracy parameters) and certain physical–mechanical properties of the printed part material (resistance to different categories of stress, hardness, etc.) can be taken into consideration.

The main groups of factors able to influence the values of such output parameters are:

- The chemical composition of the part material and some physical–mechanical properties of this material;
- The way the material is arranged inside the part, defined by parameters such as the type of raster, the thickness of the deposited layer, the infill density, etc.;
- The parameters characterizing the printing conditions (printing speed, build plate temperature, extrusion temperature, presence or absence of a cooling process, etc.).

Factors from the previously mentioned categories were considered when the question of conducting experimental research was formulated. The experimental research aimed to highlight the influence of several factors on the compression behavior of some balls manufactured by 3D printing from ceramic composite materials with a polymer matrix.

### 2.2. Modeling the Behavior of Balls Made of Ceramic–Polymer Composites under the Action of Axial Stresses

For FEM (finite-element method) results to be as accurate as possible, the material assigned to the ball sample has to mimic mechanical properties presented by the manufacturer for the filament used for 3D printing. Ansys 2023R2 (researcher license) Material Design module was used as the tool to obtain the new material. Polylactic acid was used for the matrix, and a ceramic was used for the particles. The module mentioned above allows a new material design called representative volume element (RVE). It resulted in particles of about 10 µm with no hollows inside. Mesh-wise, the adapt towards edges option with block decomposition was used to generate a block-based structured mesh. It was set to conform to the process of meshing, which implies that nodes found at interfaces are shared. Settings were established to be orthotropic for the type of anisotropy, which computes linear elasticity.

The design was later imported into the Explicit Dynamics module of Ansys. Inside the Connections branch, default body interactions were considered. The ball test sample received a Hex Dominant method of meshing that set all quads for the free face mesh type and a body sizing for the ball sample of 0.7 mm. That resulted in 28,862 elements and 23,596 nodes for the assembly. The mesh metric indicates tetrahedral elements with 4 nodes (Tet4), hexahedron elements with 8 nodes (Hex8), wedge (prism) elements with 6 nodes (Wed6), and pyramidal elements with 5 nodes (Pyr5) when assessing element quality.

**Figure 4:**
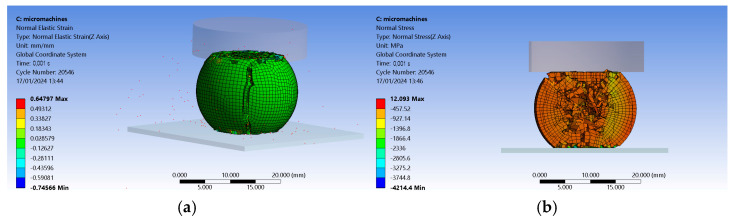
Finite-element method of the behavior of a ceramic composite ball with a polymer matrix under a compressive load: (**a**) 3D view of strain distribution; (**b**) section view of stress distribution.

Analysis settings considered a one-step-in-time of 0.001 s with automatic mass scaling. According to the experimental results, the mean value of 3.23200 kN was used as the compressive load at maximum compressive extension for the 50% concrete filament, and the table on which the ball stood was considered fixed. The results are consistent with the experimental ones. Strain registered as 0.64797 mm/mm as opposed to 0.60698 mm/mm in the experiments (see Figure 4a). Under the compressive load, the sample flattened at both ends, and the crack was similar in shape and the direction of propagation to that of the experimentally tested. Stress registered as 12.093 MPa as opposed to the value of 10.28777 MPa obtained in the experimental tests (see Figure 4b). A section view presents a detailed stress distribution as the core of the sample crumbles to pieces, thus initiating the appearance of the crack.

Further refinement may be needed in terms of mesh quality and analysis settings to obtain more appropriate values. That requires significant computing power. The authors recommend the use of the results with care.

### 2.3. The Ceramic–Polymer Composite Materials Used to Manufacture the Balls

The verification of some of the previously formulated hypotheses and the accumulation of additional information regarding the behavior of ball bodies under the action of compression stresses was carried out by manufacturing balls with a diameter of 20 mm from four distinct ceramic–polymer composites (FromFutura PLA Pottery clay, FormFutura PLA Terracotta, FormFutura PLA Granite, and FormFutura PLA Concrete; manufacturer: FormFutura, The Netherlands).

**Figure 5:**
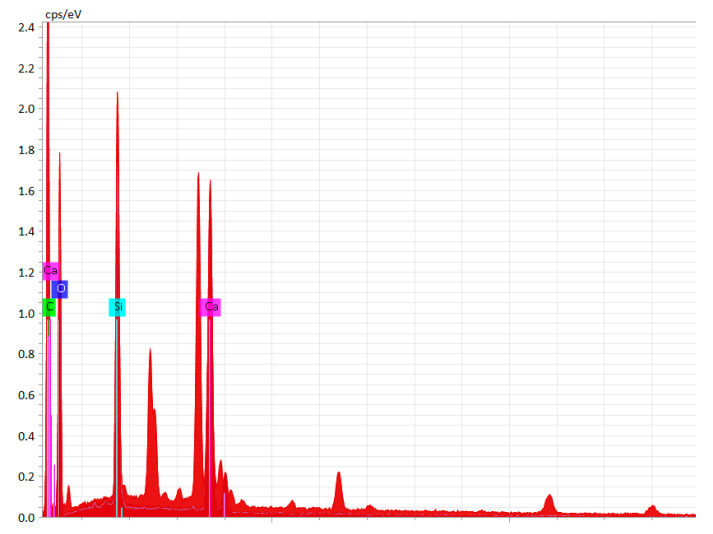
Energy-dispersive X-ray spectroscopy (EDS) spectra of a ball made of the terracotta ceramic–polymer composite material.

Some information on the chemical composition of the materials used to manufacture the balls is included in Table 1. The chemical composition of the materials from which the balls were made was determined using a scanning electron microscope (SEM), the Vega II Tescan LSH (Brno, Czech Republic). The manufacturer of the ceramic–polymer composite materials notes that roughly the same chemical composition was used for all four materials, but different dyes were added. The information in Table 1 confirms the existence of similar chemical compositions for all 4 ceramic–polymer composite materials. In Figure 5, the spectrum obtained by energy-dispersive X-ray spectroscopy (EDS) can be observed in the case of a terracotta ball.

A thermogravimetric analysis of these materials was performed to better characterize the ceramic–polymer composite materials used in manufacturing the balls. It is known that thermogravimetric analysis involves measuring the variation over time in the mass of a sample under conditions of a controlled atmosphere and controlled variation in the temperature of the environment in which the testing takes place. Thermogravimetric analysis provides additional information on the thermal phenomena associated with polymer composite materials when such materials are heated under predetermined temperatures and heating rates. In this way, some changes that affect ceramic–polymer composite materials during the 3D printing process, i.e., the manufacturing of balls, can be justified.

The thermogravimetric analyses were performed using TGA 2 equipment from Mettler Toledo (Columbus, OH, USA). Samples of PLA with concrete, granite, pottery clay, and terracotta reinforcements were heated in the equipment furnace at 10 k/min using a purge gas flow rate of 50 mL/min. At 600 °C, the purge gas was switched from nitrogen to air. The degradation of the PLA mass stars near 250 °C flowed by a significant material loss due to pyrolysis up to 480 °C.

**Figure 6:**
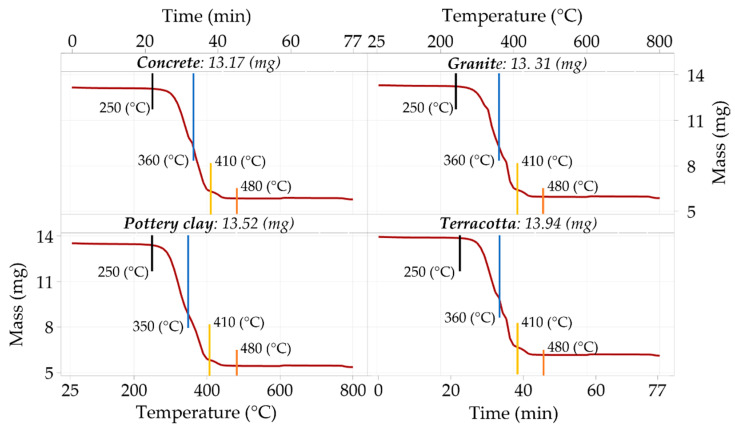
Examples of the use of thermogravimetric analysis to highlight the variation in sample masses depending on temperature (for concrete and pottery clay materials) and depending on time (for granite and terracotta materials).

**Figure 7:**
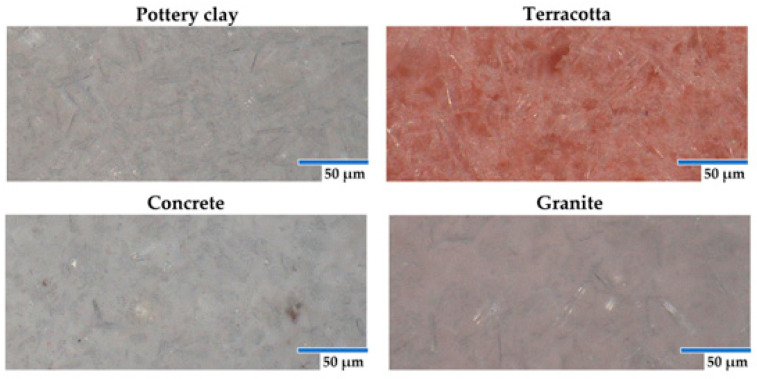
Image of PLA furnace residuals (images obtained using the Keyence Vhx-7000 digital microscope produced by Keyence-Osaka, Osaka, Japan.

The material loss was recorded in three steps, as presented in Figure 6 and Table 2. In the first step, between 280 °C and 360 °C, the samples lose a significant amount of the initial mass (Table 1). In the second step, between 360 °C and 410 °C, the samples lose up to 3 mg. These mass loss stages correspond to the PLA polymeric material’s degradation and decomposition range, from 320 °C to 390 °C [24,25,26]. In the following step, between 410 °C and 480 °C, the materials lose a small fraction of the initial mass of 0.39–0.51 mg, with a remaining residue of 39.6–44.12% (relative to the input). The range is smaller than the manufacturer’s 50% declared filling [27]. However, the resulting residuals presented no trace of carbonization even after air was introduced at 600 °C. When magnifying the residuals, it can be observed that the fillers have an aspect similar to short fibers (Figure 7).

Table 3 shows the results of tracking the change in the masses of 6 samples from the materials used during the gravimetric analysis.

Using gravimetric analysis for samples from the materials needed to manufacture spherical bodies revealed quite similar behaviors of the materials under controlled heating. The finding can be justified by the similar chemical compositions of the materials, the changes in chemical composition being determined only by adding different dyes.

### 2.4. Experimental Conditions

An Ultimaker 2+ printer (made in The Netherlands) was used to form the balls.

The compression testing of the balls manufactured by 3D printing from the four ceramic–polymer composite materials was performed on a computer-controlled electronic universal testing machine (WDW type, made in China). The compression speed was 5 mm/min in all experimental tests.

Experimental research was considered to highlight the influence exerted by factors characterizing the printing conditions on the limit of elastic resistance to the deformation of test samples in the form of balls made of different ceramic–polymer composite materials subjected to compression stresses.

**Figure 8:**
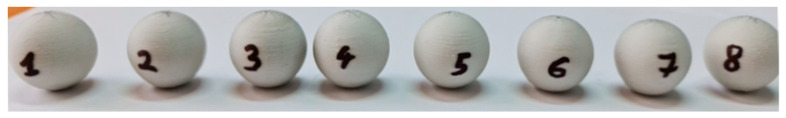
Concrete ceramic composite material specimens before their compression testing.

**Figure 9:**
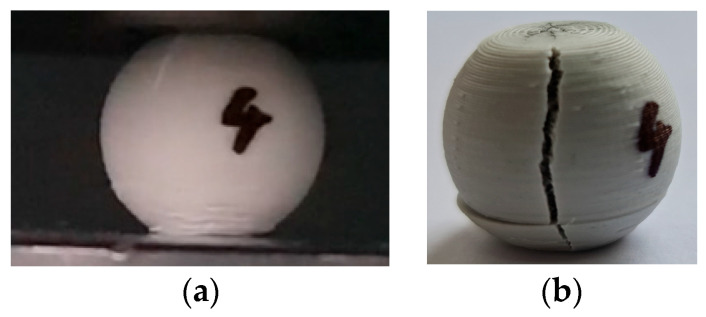
The moment when, on the upper left, a first crack appears (**a**) and the moment when the testing was stopped (**b**) in the case of experiment no. 4, performed on a sample of the ceramic composite material with the concrete polymer matrix.

The hypothesis was accepted that for the ranges of variation of the 7 variables, the output parameter (the value of the maximum force corresponding to the elastic deformation limit) would have a monotonic variation, which would allow the experimental tests to be carried out for two levels of variation of the input factors. A fractional factorial experiment of the Taguchi L8 type (2^8−1^) was used to diminish the number of experimental tests required to be performed [28,29]. According to such a program, it is necessary to carry out 8 experimental tests on 8 balls of the same material, with 7 input factors at two levels of variation. An image of the 8 balls of the concrete-type ceramic composite material before they were subjected to compressive stresses can be seen in Figure 8. It is necessary to mention that the balls present a small, flat surface in the lower area generated by the printing process. Currently, it is difficult or even impossible to avoid the appearance of this small, flat surface when using the FFF process. The appearance and propagation of a first crack due to ball compression is illustrated in Figure 9a.

As mentioned, the maximum value of the force, F, was taken into account as the output parameter, upon which the sudden decrease in the magnitude of the force was recorded as a result of the initiation and development of the cracking process.

In the conditions mentioned earlier, the printing speed (*v_min_* = 30 mm/s, *v_max_* = 50 mm/s), the thickness of the printed layer (*t_min_* = 0.15 mm, *t_max_* = 0.2 mm), the infill density (*i_min_* = 50%, *i_max_* = 100%), the build plate temperature (*θ_p min_* = 50 °C, *θ_p max_* = 60 °C), the extrusion temperature (*θ_e min_* = 215 °C, *θ_e max_* = 230 °C), the presence (*c*_1_) or absence of cooling (*c*_2_), and the raster angle (*α_min_* = 45°, *α_max_* = 90°) were considered as input factors.

## 3. Results

The experimental results and the values of some of the input factors in the process of 3D printing of the ceramic composite balls with a polymer matrix are included in Table 4.

The previously accepted hypothesis that, for the ranges of variation in the values of the input factors, the value of the output parameter will have a monotonous variation, so without maxima or minima, allows the use of empirical mathematical models of the power function type for modeling the variation in the elasticity limit with respect to the values of the input factors in the printing process.

The mathematical processing of the experimental results was carried out using the Minitab software, created in Machine Manufacturing Technology Department, from Gheorghe Asachi Tehnical University of Iasi by one of the proffesors from the department.

The assessment of the degree of adequacy of the identified mathematical models can be realized by analyzing the values of the coefficients of determination, *R*^2^, which are also calculated using the Minitab software.

Under the conditions mentioned earlier, the following empirical mathematical models of the power-type function were identified:

- For the pottery clay-type material:

*F* = 0.0985*v*^0.0212^*t*^0.105^*i*^0.632^*θ_p_*^−0.0401^*θ_e_*^0.255^*c*^−0.001063^*r*^−0.00970^ (3)

(the value of the coefficient of the determination being *R*^2^ = 99.98);

- For the terracotta-type material:

*F* = 0.0639*v*^0.198^*t*^−0.350^*i*^0.587^*θ_p_*^0.526^*θ_e_*^−0.248^*c*^−0.0094^*θ_r_*^−0.100^ (4)

(the value of the coefficient of the determination being *R*^2^ = 98.64);

- For the concrete-type material:

*F* = 3403.831*v*^−0.272^*t*^−0.491^*i*^0.812^*θ_p_*^−0.773^*θ_e_*^−1.098^*c*^−0.260^*r*^−0.204^ (5)

(the value of the determination coefficient being *R*^2^ = 99.89);

- For the granite-type material:

*F* = 17.461*v*^−0.0728^*t*^0.0907^*i*^0.764^*θ_p_*^−0.112^*θ_e_*^−0.704^*c*^0.0254^*r*^0.0332^ (6)

(the value of the coefficient of the determination being *R*^2^ = 93.02%).

**Figure 10:**
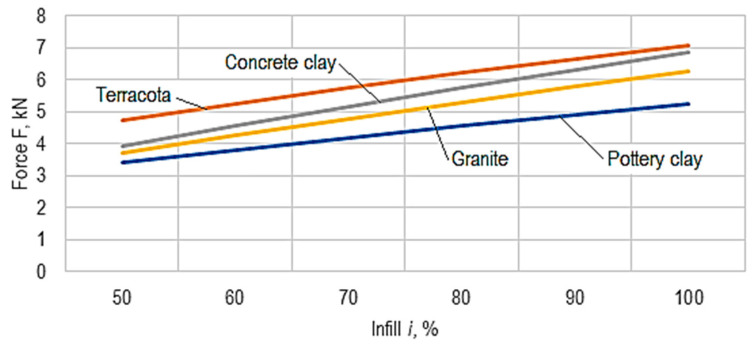
The influence of the value of infill, *I*, on the magnitude of the force, *F*, at which the ball cracks (*v* = 30 mm/s, *t* = 0.15 mm, *θ_p_* = 50°, *θ_e_* = 230°, *c* = 2, *r* = 45°).

**Figure 11:**
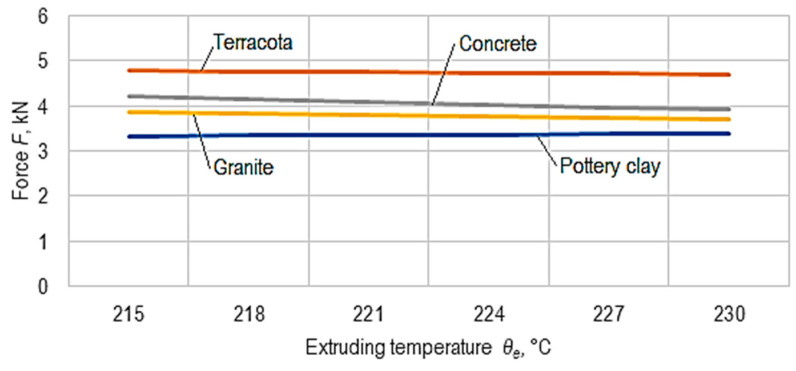
The influence of the value of the extrusion temperature, *θ_e_*, on the magnitude of the force, *F*, at which ball cracking occurs (*v* = 30 mm/s, *t* = 0.15 mm, *i* = 50%, *θ_p_* = 50 °C, *c* = 2, *r* = 45°).

**Figure 12:**
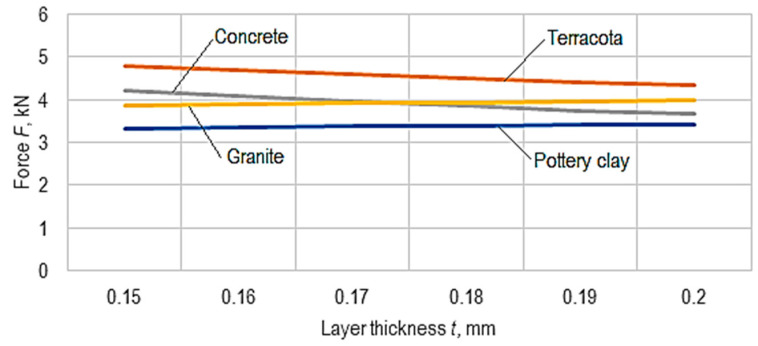
The influence of the variation in the thickness, *t*, of the deposited layer on the magnitude of the force, *F*, at which the ball cracks (*v* = 30 mm/s, *i* = 50%, *θ_p_* = 50 °C, *θ_e_* = 215 °C, *c* = 2, *r* = 45°).

Using the empirical mathematical models defined by Equations (3)–(6), the diagrams in Figure 10, Figure 11 and Figure 12 were developed.

The analysis of the empirical mathematical models and the graphical representations in Figure 10, Figure 11 and Figure 12 allowed the formulation of the following findings.

The analysis of Equations (3)–(6) highlights the fact that the values of the coefficients and the exponents attached to the input factors considered have quite different values, as well as that the meanings of their manifestation are distinct, since the exponents have both positive as well as negative values. For this reason, the common aspects corresponding to the reflection by Equations (3)–(6) of the compression behavior of the balls manufactured by 3D printing from ceramic composite materials with a polymer matrix will be considered first.

It was thus found that, as expected, the strongest influence on the force, *F*, at which the ball cracking process is initiated is exerted by the infill, *i*, factor. As the infill, *i*, value increases, in all cases, there is an increase in force *F* (Figure 10), given the fact that there is an increase in resistance to the action of force *F*, due to a greater filling of the ball with material (the presence of a larger amount of composite ceramic material in the ball).

The diagram in Figure 10 highlights that, from the point of view of the force value required for the appearance of the first crack, the ceramic composite materials with a polymer matrix are arranged in ascending order in the following way: pottery clay, granite, concrete, and terracotta. This means that the highest mechanical strength among the ceramic composite materials corresponds to the terracotta-type ceramic composite material.

Another observation concerns the minimal values of factor *c* (cooling conditions) for three of the materials used in manufacturing the balls. This means that this factor practically does not influence the magnitude of the force, *F*. The statement is, to a lesser extent, valid for the concrete-type material. Still, in this case, it was found that, compared to the other factors, the influence exerted by the cooling conditions, *c*, is the lowest, since the exponent attached to this factor has a lower value than the exponent values for the other factors.

Except for the pottery clay material, the increase in the extruding temperature, *θ_e_*, reduced the force, *F*, the most significant reduction occurring in the case of the concrete-type material (Figure 11). An explanation of this reduction could be connected to a deformation less favorable to the mechanical resistance of the wire deposited to constitute each layer in the case of the 3D printing process when the temperature, *θ_e_*, increases for three of the ceramic composite materials from which the balls were made. An inverse behavior (an increase in force, *F*, with increasing temperature, *θ_e_*) was observed in the pottery clay-type ceramic composite material, which showed the lowest mechanical resistance (Figure 11).

For three materials (an exception being the terracotta-type material), the increase in the layer thickness causes a decrease in the force, *F*, more significant in the case of concrete and less in the case of pottery clay (Figure 12).

The analysis of the appearance of the force–deformation variation diagrams highlights the fact that if, in the case of most materials, this variation has the approximate form of a straight line, in the case of the concrete-type ceramic–polymer composite material, it is found that a chain of several segments of curved lines replaces the straight line. This could mean that multiple successive local failures occur in the material of the concrete-type ceramic–polymer composite ball under compression. These failures are expected to occur at the contact of the ceramic granules with the polymer material in which the granules are embedded.

The empirical mathematical models determined and the diagrams developed based on them highlight the largely similar behavior of the ceramic–polymer composite materials. The finding can be justified if the chemical compositions of the four materials are considered; basically, they started from a single ceramic–polymer composite to which different colorants were added to obtain the appearance of known ceramic materials.

A possible use of balls made of ceramic–polymer composite materials would be as bearings or linear guides subject to low mechanical stresses. Currently, there are also balls made of ceramic materials with high resistance to mechanical stress which are used as some categories of bearings.

## 4. Conclusions

Manufacturing and studying the behavior of balls made of composite ceramic materials with a polymer matrix could be important for those practical situations where it is necessary to use spherical parts. The theoretical study of the behavior of a ball under compression highlights the possibility of some first cracks in the contact areas of the ball with the support surface and with the surface of the part through which the ball is subjected to compressive force. Finite-element modeling of such a situation can reveal the distribution of compressive stresses inside a ball. To highlight the influence exerted by different factors on the value of the force at which the ball fails by cracking, experimental research was carried out on balls with a diameter of 20 mm manufactured by 3D printing from four ceramic composite materials with a polymer matrix. The four materials were pottery clay-type, terracotta-type, concrete-type, and granite-type materials. These materials have fairly similar chemical compositions, starting from the same ceramic–polymer composite material but with different dyes being used to reach the four mentioned materials. The gravimetric analysis of some samples made of the respective materials proved a similar behavior from the point of view of the effects of applying controlled heating. The experiments were performed according to the requirements of a Taguchi fractional factorial experiment of type *L*_8_ = 2^8−1^, with seven independent variables at two levels of variation. Empirical power-function mathematical models were determined, highlighting the influence of print speed, layer thickness, infill, build plate temperature, extrusion temperature, cooling conditions, and raster angle on the compression force at which the first cracks appear. It was found that in the cases of all four composite ceramic materials with a polymer matrix, the infill exerts the strongest influence, which leads to an increase in the force that produces the initiation of some first cracks. It can be seen that the highest mechanical resistance corresponds to the terracotta-type ceramic composite material, while the concrete-type ceramic composite material has the lowest mechanical resistance. The order of ceramic composite materials with a polymer matrix from the point of view of decreasing the magnitude of the force at which the generation of a first crack occurs is the concrete type, the terracotta type, the granite type, and the pottery clay type. In the future, the experimental research is intended to expand by considering other ceramic–polymer composite materials used in the 3D printing of ball-type parts and other factors whose variation could change the force value at which the ball cracking process begins.

## Figures and Tables

**Table 1:** The percentage contents of the main elements in the four ceramic–polymer composite materials used in the 3D printing of balls.

Element	Pottery Clay	Terracotta	Concrete Clay	Granite
Carbon (%)	49.6819	50.69737	51.58114	51.10442
Oxygen (%)	40.89724	40.59158	44.00292	45.53762
Calcium (%)	5.934304	5.297653	2.792067	2.085428
Silicon (%)	3.48655	3.413395	1.623869	1.272532

**Table 2:** Stages of mass loss of composite ceramic samples during thermogravimetric analysis.

	Input (mg)	Step 1 (mg)	Step 2 (mg)	Step 3 (mg)	Rest (mg)
Pottery clay	13.5200	4.8774	2.8189	0.3914	5.3550
Terracotta	13.9400	4.2861	2.9841	0.5128	6.1060
Concrete	13.1700	4.0588	2.8036	0.4596	5.7940
Granite	13.3100	4.0176	2.8733	0.4658	5.4730

**Table 3:** The average mass of the resulting samples of R1 (50% infill) and R3 (100% infill) printed samples (Sartorius BP 221 S analytical balance).

Ceramic–Polymer Composite	Infill (%)	Mass (g) for the Resulting Test Sample No.						Mean Mass (g)	Standard Deviation
		s1	s2	s3	s4	s5	s6		
Pottery clay	50	4.8206	4.7941	4.8577	4.8239	4.9086	4.8936	4.8498	0.0449
	100	6.6832	6.6571	6.6855	6.6818	6.6725	6.7068	6.6812	0.0163
Terracotta	50	4.4664	4.3893	4.4050	4.4624	4.3859	4.4940	4.4338	0.0461
	100	6.0915	6.0566	6.0821	6.0554	6.1112	6.0729	6.0783	0.0214
Concrete	50	4.4110	4.4191	4.3790	4.4788	4.4683	4.4427	4.4332	0.0375
	100	5.6161	5.6565	5.6663	5.6716	5.6140	5.6463	5.6451	0.0249
Granite	50	4.4987	4.4520	4.4551	4.4712	4.4184	4.5055	4.4668	0.0324
	100	6.2217	6.2030	6.1936	6.1858	6.1661	6.2049	6.1959	0.0189

**Table 4:** Experimental conditions and results.

	Values of the Input Factors							Values of the Output Parameter (Maximum Force, *F* (kN))			
Part/ Exp No.	Printing Speed, *v* (mm/s)	Layer Thickness, *t* (mm)	Infill Density, *i* (%)	Temperature of the Build Plate, *θ_p_* (°C)	Extruding Temperature, *θ_e_* (°C)	Cooling Condition, *c*	Raster Angle, *r* (°)	Pottery Clay	Terracotta	Concrete	Granite
1	30	0.15	50	50	230	2	45	3.99	3.39	3.90	3.65
2	30	0.15	50	60	215	1	90	3.87	3.56	3.79	3.77
3	30	0.2	100	50	230	1	90	6.52	4.32	6.18	6.4
4	30	0.2	100	60	215	2	45	6.40	5.15	5.56	6.54
5	50	0.15	100	50	215	2	90	6.28	5.34	5.57	6.41
6	50	0.15	100	60	230	1	45	6.39	6.24	6.20	5.75
7	50	0.2	50	50	215	1	45	4.09	3.47	3.80	3.72
8	50	0.2	50	60	230	2	90	4.10	3.48	2.22	3.62

Cooling condition: 1—without cooling, 2—with cooling.

## Data Availability

Data are contained within the article.
